# Natural Honey and Cardiovascular Risk Factors; Effects on Blood Glucose, Cholesterol, Triacylglycerole, CRP, and Body Weight Compared with Sucrose

**DOI:** 10.1100/tsw.2008.64

**Published:** 2008-04-20

**Authors:** N. Yaghoobi, Noori Al-Waili, M. Ghayour-Mobarhan, S. M. R. Parizadeh, Z. Abasalti, Z. Yaghoobi, F. Yaghoobi, H. Esmaeili, S. M. R. Kazemi-Bajestani, R. Aghasizadeh, Khelod Y. Saloom, G. A. A. Ferns

**Affiliations:** ^1^ Heart and Vascular Research Center, Bu-Ali Research Institute, Mashhad University of Medical Science, Mashhad, Iran; ^2^ Department of Nutrition and Biochemistry, Faculty of Medicine, Mashhad University of Medical Sciences, Mashhad, Iran; ^3^ AL-Waili's Foundation for Science and Trading, New York, NY, USA; ^4^ Khorasan Health Center, Mashhad University of Medical Science, Mashhad, Iran; ^5^ Department of Statistics, Faculty of Medicine, Mashhad University of Medical Sciences, Mashhad, Iran; ^6^ Centre for Clinical Science and Measurement, University of Surrey, Stag Hill, Guildford, Surrey, UK

**Keywords:** honey, body weight, cholesterol, HDL-C, LDL-C, CRP, triacylglycerole

## Abstract

It has been found that honey ameliorates cardiovascular risk factors in healthy individuals and in patients with elevated risk factors. The present study investigated the effect of natural honey on total cholesterol, low-density lipoprotein cholesterol (LDL-C), high-density lipoprotein cholesterol (HDL-C), triacylglycerole, C-reactive protein (CRP), fasting blood glucose (FBG), and body weight in overweight individuals. There were 55 patients, overweight or obese, who were randomly recruited into the study and assigned into two groups: control group (17 subjects) and experimental group (38 subjects). Patients in the control group received 70 g of sucrose daily for a maximum of 30 days and patients in the experimental group received 70 g of natural honey for the same period. In the control and experimental groups, body weight, body mass index, body fat weight, total cholesterol, LDL-C, HDL-C, triacylglycerole, FBG, and CRP were measured before treatment and at day 31 after the commencement of treatment. Results showed that honey caused a mild reduction in body weight (1.3%) and body fat (1.1%). Honey reduced total cholesterol (3%), LDL-C (5.8), triacylglycerole (11%), FBG (4.2%), and CRP (3.2%), and increased HDL-C (3.3%) in subjects with normal values, while in patients with elevated variables, honey caused reduction in total cholesterol by 3.3%, LDL-C by 4.3%, triacylglycerole by 19%, and CRP by 3.3% (*p* < 0.05). It is our conclusion that consumption of natural honey reduces cardiovascular risk factors, particularly in subjects with elevated risk factors, and it does not increase body weight in overweight or obese subjects.

### INTRODUCTION

Honey has been used for as long as there has been medicine. The ancient Sumerians, Babylonians, and Egyptians all recorded its use, e.g., in Sumerian clay tablets estimated to be 4,000 years old and in Egyptian papyri dated from 1900–1250 B.C. The ancient Egyptians used honey in embalming and made salves with it for treating diseases of the eye and skin.; honey was used as a drug more than a nutrient. Honey was valued highly in the Middle East and was mentioned in the Holy Quran 1,400 years ago. ("And the LORD taught the bee to build its cells in hills, on tree and in men's habitations, then to eat of all the produce of the earth and find with skill the spacious paths of its LORD, there issues from within their bodies a drink of varying colors, wherein is healing for men, verily in this is a sign for those who give thought.") It was also mentioned in the Holy Talmud and Holy Bible. Avicenna (Ebne Sina), the famous Persian philosopher and physician about 1,000 years ago, mentioned the therapeutic effect of honey in *Ghanoon*, his published book[1].

There are over 300 types of floral honey available in the marketplaces of the world. The color, flavor, and mineral and vitamin content of honey depend on the flower from which bees gather the nectar. In modern medicine, honey has been used for the treatment of respiratory disease, urinary disease, gastrointestinal disease, skin ulcers, wounds, eczema, psoriasis, dandruff, diaper dermatitis, and radiation mucositis[2,3,4,5,6,7,8]. Laboratory studies and clinical trials have shown that honey shows a broad-spectrum antimicrobial activity[9,10,11,12,13], and increases antibody production against thymus-dependent and thymus-independent antigens[14]. Consumption of honey decreases the concentrations of prostaglandins in the plasma of normal individuals[15], and increases antioxidant agents, serum iron, and blood indices[16]. Honey increases insulin secretion and decreases blood glucose levels[17,18,19]. Al-Waili found that natural honey also improves lipid profile, lowers normal and elevated C-reactive protein (CRP), lowers homocysteine, and decreases triacylglycerole in patients with hypertriglyceridemia[19]. The ability of honey to modulate total cholesterol, low-density lipoprotein cholesterol (LDL-C), high-density lipoprotein cholesterol (HDL-C), triacylglycerole, homocysteine, and CRP carries a potential role of using honey to reduce cardiovascular risk factors. Honey contains glucose and fructose. Fructose or glucose affects total cholesterol, triacylglycerole, and other risk factors. The purpose of this study is to investigate the effect of natural honey collected from Iran on cardiovascular risk factors, such as total cholesterol, LDL-C, HDL-C, triacylglycerole, CRP, and body weight, in overweight or obese subjects with normal or elevated cardiovascular risk factors; sucrose was used as control because it is commonly used as a sweetener and it contains glucose and fructose.

### MATERIALS AND METHODS

#### Subjects

There were 60 overweight or obese subjects (body mass index [BMI] >25 kg/m$^2$), aged between 20 and 60 years, who were randomly recruited from the educational department of Mashhad University of Medical Science. Subjects were randomly allocated to either the experimental group (n = 40) or a control group (n = 20). Subjects who were <20 or >60 years old were excluded from the study, as were subjects who used any medications. Among them, 55 subjects remained in either the experimental group (n = 38, age 42.6 ± 8.6 years) or the control group (n = 17, age 39.6 ± 10.6 years). There were 24 males and 31 females, and the mean age of subjects was 41.2 ± 9.2 years.

Subjects in the control group received 70 g of sucrose dissolved in 250 ml tap water daily, while subjects in the experimental group received 70 g of honey dissolved in 250 ml tap water each day for a maximum of 30 days. Subjects in both groups did not undergo a special diet regimen, drug therapy, or change in their lifestyle, such as physical exercise, during the period of study. Natural unprocessed honey was used for experimentation.

The subjects were followed and any side effects on using the interventions were recorded. Any new symptom that appeared after commencement of intervention intake was considered a side effect and recorded.

Each subject gave informed written consent to participate in the study, which was approved by the Mashhad University of Medical Science Ethics Committee.

### Anthropometric Measurements

Anthropometric parameters, including weight, height, and waist circumference, were determined before and after intervention using a standard protocol. Subjects were asked to breathe normally and to breathe out gently at the time of the measurement. Height and body weight were measured with the subjects dressed in light clothing after an overnight fast. The body weight of each subject was measured with a standard scale to an accuracy of ±0.1 kg and height was measured to an accuracy of ±0.1 cm. The BMI was calculated as weight (kg) divided by height squared ($m^2$).

Body fat percentage (BF%) and body fat weight (BFW) were measured before interventions and repeated after 30 days. BF% and BFW were measured by a hand-held Bio impedance analyzer with the CV of less than 1%.

### Routine Biochemical Analysis

Blood samples were collected in the morning after an overnight fast from each subject. Laboratory data, including full fasting lipid profile comprising total cholesterol and HDL-C, LDL-C, triacylglycerole, and fasting blood glucose (FBG), were determined for each patient. All of the above were measured by routine methods. Hs-CRP was measured by a high-sensitivity PEG-enhanced immunoturbidimetry method with an Alycon analyzer (ABBOTT, Chicago, IL). The within assay and between assay precision was less than 1% for all biochemical assays. These variables were repeated 30 days after commencement of intervention intake.

### Statistical Analysis

Statistical analysis was undertaken with the use of MINITAB software (release 13; Minitab Inc., State College, PA). Paired *t*-test to compare between means before and after intervention in the same group and unpaired *t*-test to compare between control and honey group were used for statistical analysis; $p < 0.05$ was considered as statistically significant. Categorical data were compared by using Fisher's exact test or chi-square test. Analysis of covariance was used to assess differences after adjustment for important confounding factors, such as age and physical activity.

## RESULTS

There were no statistically significant differences in the mean values of age, BMI, BF%, lipid profiles, and fasting blood glucose (FBG) between the control and experimental groups ($p > 0.05$, Tables 1 and 2). However, the sucrose group was significantly heavier than the honey group; they were approximately 10 kg heavier than the experimental group ($p < 0.05$, Table 1). Tables 1 and 2 demonstrate the effects of sucrose or honey intake on body weight, body fat, lipid profile, FBG, and CRP. The results showed that honey caused significant reduction of BMI (1.14%, $p = 0.02$) and FBG (4.2%, $p = 0.04$) in subjects with normal values (Tables 1 and 2). Also, honey significantly reduced serum triacylglycerole (19%, $p = 0.006$) and hs-CRP (3.3%, $p = 0.008$) in subjects with elevated variables (Tables 1 and 2). However,

**TABLE 1**
**Effects of Sucrose and Honey on Anthropometric Measurements and Serum Triacylglycerole**

| | **Age (Years)** | | **Weight (kg)** | | **BF%** | | **Weight Fat (kg)** | |
|---|---|---|---|---|---|---|---|---|
| Type of treatment | S | H | S | H | S | H | S | H |
| Number | 17 | 38 | 17 | 38 | 17 | 38 | 17 | 38 |
| Before | 42.4 ± 8.7 | 39.6 ± 10.6 | 87.6 ± 11.3 | 77.4 ± 9.4 | 32.4 ± 9.9 | 34.0 ± 6.8 | 28.5 ± 10.9 | 26.3 ± 5.9 |
| After | — | — | 88.1 ± 11.7 | 76.4 ± 9.2 | 32.7 ± 9.4 | 33.4 ± 7.5 | 29.3 ± 10.7 | 26.0 ± 7.5 |
| *p* Value | — | — | 0.07 | 0.09 | 0.511 | 0.279 | 0.175 | 0.682 |
| Changes (%) | — | — | ↑ 0.6 | ↓1.3 | ↑0.9 | ↓1.8 | ↑2.8 | ↓1.1 |

| | **BMI (kg/m$^2$)** | | **Triacylglycerole (mg/dl)** | | **Triacylglycerole >150 mg/dl** | |
|---|---|---|---|---|---|---|
| Type of treatment | S | H | S | H | S | H |
| Number | 17 | 38 | 17 | 38 | 10 | 15 |
| Before | 32.6 ± 6.6 | 30.1 ± 3.1 | 183 ± 108 | 156 ± 80 | 250 ± 110 | 237 ± 56 |
| After | 32.8 ± 5.0 | 29.8 ± 3.2 | 182 ± 132 | 139 ± 67 | 240 ± 162 | 192 ± 59 |
| *p* Value | 0.199 | 0.02 | 0.988 | 0.171 | 0.801 | 0.006 |
| Changes (%) | ↑0.4 | ↓1.2 | ↓0.6 | ↓11 | ↑↓4 | ↓19 |

*Note:* Values are expressed as Mean ± SD, or percentages. Comparison between pre- and post-treatment were assessed by paired t-test. T-test was used for comparison between pretreatment honey and sucrose subjects, and only sucrose group was significantly heavier than honey group ($p < 0.05$). S = sucrose; H = honey.

**TABLE 2**
**Effects of Sucrose and Honey on Biochemical Markers**

| | **Hs-CRP (mg/dl)** | | **Hs-CRP >5 mg/dl** | | **LDL-C (mg/dl)** | | **LDL-C >130 (mg/dl)** | |
|---|---|---|---|---|---|---|---|---|
| Type of treatment | S | H | S | H | S | H | S | H |
| Number | 17 | 38 | 3 | 17 | 17 | 38 | 6 | 21 |
| Before | 4.8 ± 3.2 | 6.3 ± 4.2 | 9.9 ± 3.6 | 9.8 ± 2.8 | 127 ± 40 | 137 ± 46 | 165 ± 11.8 | 164 ± 28 |
| After | 4.7 ± 3.1 | 6.1 ± 4.1 | 9.8 ± 3.1 | 9.5 ± 2.8 | 130 ± 49 | 129 ± 53 | 180 ± 30 | 157 ± 37 |
| p Value | 0.421 | 0.28 | 0.89 | 0.008 | 0.671 | 0.062 | 0.216 | 0.204 |
| Changes (%) | ↓ 2 | ↓ 3.2 | ↓ 1 | ↓ 3.3 | ↑ 2.4 | ↓ 5.8 | ↑ 9 | ↓ 4.3 |

| | **TC (mg/dl)** | | **TC >200 (mg/dl)** | | **HDL-C (mg/dl)** | | **FBG (mg/dl)** | |
|---|---|---|---|---|---|---|---|---|
| Type of treatment | S | H | S | H | S | H | S | H |
| Number | 17 | 38 | 13 | 30 | 17 | 38 | 17 | 38 |
| Before | 220 ± 39 | 226 ± 50 | 237 ± 25 | 242 ± 39 | 56 ± 13 | 60 ± 14.1 | 93.2 ± 14.7 | 96.2 ± 44.2 |
| After | 223 ± 46 | 219 ± 57 | 239 ± 42 | 234 ± 52 | 58 ± 15 | 62 ± 14.8 | 95.3 ± 18.4 | 92.2 ± 39.2 |
| p Value | 0.368 | 0.165 | 0.844 | 0.137 | 0.308 | 0.40 | 0.341 | 0.042 |
| Changes (%) | ↑ 1.4 | ↓ 3 | ↑ 0.8 | ↓ 3.3 | ↑ 3.6 | ↑ 3.3 | ↑ 2.2 | ↓ 4.2 |

*Note:* Values are expressed as Mean ± SD, or percentages. Comparison between pre- and post-treatment were assessed by paired t-test. T-test was used for comparison between pretreatment honey and sucrose subjects and no significant differences were observed ($p > 0.05$). S = sucrose; H = honey; Hs-CRP = high-sensitivity C-reactive protein; LDL-C = low-density lipoprotein cholesterol; TC = total cholesterol; HDL-C = high-density lipoprotein cholesterol; FBG = fasting blood glucose.

honey consumption had a nonsignificant mild reduction in body weight (1.3%), BF% (1.8%), and BFW (1.1%) ($p > 0.05$, Table 1). Honey nonsignificantly reduced total cholesterol (3%), LDL-C (5.8%), triacylglycerole (11%), and CRP (3.2%), and increased HDL-C (3.3%) in subjects with normal values (*p*

> 0.05), while in subjects with elevated variables, honey caused a reduction in total cholesterol by 3.3% and LDL-C by 4.3% ($p > 0.05$, Tables 1 and 2).

No significant changes were found in anthropometric measurement and biochemical markers within the control group pre- and postintervention. No side effect was reported with use of either intervention.

### DISCUSSION

The main finding of this study was the ability of natural honey to modulate some of the cardiovascular risk factors. Although some results were statistically insignificant, they might be clinically important and they could be statistically significant when honey is used for a longer period. Present results are substantiated by previous work showing that honey could ameliorate cardiovascular risk factors after 2 weeks of commencement of its ingestion[19]. This is extremely important, not only because cardiovascular diseases are a leading cause of death beside cancer, but using honey is a novel approach because honey is a natural nutritional material. Honey could provide the body with invaluable nutritional ingredients as well as antioxidant substances. Basically, the mechanism of action of honey is not clear; however, honey contains many trace elements, such as copper, zinc, antioxidants, and unidentified materials. Its content of fructose and glucose might have an important role in its effect.

Natural honey decreased total cholesterol and LDL-C in overweight healthy and overweight hyperlipidemic subjects. Increasing dietary fructose from 3 to 20% of calories at the expense of starch increased total cholesterol by 9% and LDL-C by 11%[20]. Therefore, sucrose might increase lipids because of the presence of fructose. Honey contains fructose, but it decreased blood lipid levels. The difference between the effects of sucrose and natural honeys on cholesterol might be due to the presence of certain substances in natural honey[19].

Cardiac risk factors, such as obesity, smoking, hypertension, and chronic periodontal disease, are associated with elevated CRP levels. CRP may be involved in the pathogenesis of atherothrombosis[21]. CRP also decreased endothelial nitric oxide (NO) release and this effect remained unchanged by hyperglycemia. Through decreasing NO synthesis, CRP may facilitate the development of diverse cardiovascular diseases[22]. In the present study, honey reduced CRP, a marker of inflammation. Al-Waili reported for the first time that oral honey could reduce plasma and urinary PGE2, PGF2-alpha, and thromboxane B2[23]. Therefore, it appears that honey has anti-inflammatory properties that make it a suitable nutrient to be used in acute or chronic inflammatory conditions[19]. Many reports have demonstrated lowering effects of antioxidants on CRP levels[24,25]. Honey contains many antioxidants; therefore, honey might reduce CRP by its antioxidant properties[19].

High-carbohydrate/low-fat, isocaloric diets have been shown to increase plasma triglyceride concentrations. Fructose feeding resulted in hyperinsulinemia and elevated blood pressure when compared with that in controls[26]. Both chronic and acute consumption of large amounts of fructose are reported to elevate triglyceride levels[27,28]. However, low doses of fructose added to glucose caused significant decline in triglyceride levels[28]. It was found that a fructose diet had no persistent effect on total cholesterol, HDL-C, or LDL-C[29]. In addition, a hypertriglyceridemic effect of fructose was not observed when fructose was provided by honey in rats[28]. Repeated intravenous infusion of honey reduced FBG, and intravenous administration of honey slowly or rapidly caused lower hyperglycemia than that obtained with use of dextrose[30]. This might be due to stimulation of insulin secretion, stimulation of glucose liver uptake by glucokinase, and presence of fructose with glucose that might reduce hyperglycemic response of the glucose content of honey. Significant reduction of triglyceride levels was observed after acute blood loss when rats were fed honey[31].

Honey increased the serum level of zinc and copper that is important for insulin and glucose metabolism[16]. Glucose tolerance testing demonstrated improved glucose tolerance in a high-fructose diet, which may be related to increased insulin receptor substrate-2 expression[32]. In normal adults and adults with type 2 diabetes, a small dose of fructose administered with glucose decreased the glycemic response to a glucose load[33,34]. Low-dose fructose improved the glycemic response to an oral glucose load in normal adults without significantly enhancing the insulin or triglyceride response[33]. It was found that a small amount of fructose could increase hepatic glucose uptake and glycogen storage, and reduce glycemia and insulin level in dogs[35]. In addition, honey could reduce prostaglandin levels and elevate NO. It was demonstrated that PGE2 is one of the major physiological inhibitors of insulin, acting via specific receptors[36]. IL-1beta and PGE2 are two well-recognized inhibitors of glucose-induced insulin secretion and endogenous PGE2 mediates the inhibitory effects of IL-1beta on beta-cell function[37]. In addition, different NO donors stimulated a marked increase in insulin secretion[38]. Therefore, honey might partly affect glucose levels through its effects on prostaglandin and NO production.

Honey may have a fantastic effect on modifying the traditional cardiovascular risk factors. Specifically, the role of honey in weight reduction, which can be confirmed with prospective huge sample studies, would be attractive. These present findings encourage the use of honey for a longer term to see its effect over time and pave the way for larger randomized controlled studies.

### CONCLUSION

Consumption of natural honey in overweight and obese subjects reduced cardiovascular risk factors, particularly in subjects with elevated risk factors, and it did not increase body weight in overweight or obese subjects. The cardioprotective effects of honey may be partly explained by its anti-inflammatory effect.

### ACKNOWLEDGMENTS

This research project has been supported by the Mashhad University of Medical Science Research Council. We express appreciation to the staff of the educational department of Mashhad University of Medical Science. The participation of the staff of Avicenna (Bu-Ali) Research Institute of Mashhad University of Medical Science is also gratefully acknowledged.
